# Effect of Tea Polyphenol on Oxidative Injury in S180 Cells Induced Hepatocarcinoma Mice

**DOI:** 10.3390/ijms13055571

**Published:** 2012-05-09

**Authors:** Bo-Kang Cui, Su Liu, Shu-Hong Li, Jun Wang, Qi-Bo Wang, Sheng-Ping Li, Xiao-Jun Lin

**Affiliations:** 1 Department of Hepatobiliary Oncology, Cancer Center, Sun Yat-sen University, 651 Dongfeng East Road, Guangzhou 510060, China; E-Mails: cuibk@sysucc.org.cn (B.-K.C.); lishh@sysucc.org.cn (S.-H.L.); wangjun@sysucc.org.cn (J.W.); wangqb@sysucc.org.cn (Q.-B.W.); lishp@sysucc.org.cn (S.-P.L.); 2 State Key Laboratory of Oncology in Southern China, Guangzhou 510060, China; 3 Department of Pharmocology, Guangzhou Institute for Drug Control, 23 Xizeng Road, Liwan District, Guangzhou 510160, China; E-Mail: sliugzzs@163.com

**Keywords:** S180 cell, antioxidant, AST, mice, tea polyphenol, hepatocarcinoma

## Abstract

The purpose of this study was to evaluate the antioxidant nature of tea polyphenol on S180 cells induced liver cancer in mice. In the present study, hepatocellular carcinoma was induced by tumor transplantation of liver *in situ*. The antitumor activity of tea polyphenol has been determined *in vivo* in hepatocellular carcinoma mice after treatment of drug (50, 100, 150 mg/kg body weight) by gavage for 20 days. Results showed that a significant increase in serum aspartate transaminase (AST), alkaline phosphatase (ALP), alanine aminotransfere (ALT), malondialdehyde (MDA) level, decrease in serum white blood cells (WBC), serum total protein (TP), albumin (ALB), A/G, tumor necrosis factor-α (TNF-α) and interferon-gamma (IFN-γ), liver reduced glutathione (GSH) levels were observed. In addition, the levels of enzymic and non-enzymic antioxidants were decreased when subjected to S180 cells induction. These altered enzyme levels were ameliorated significantly by administration of tea polyphenol at the concentration of 50, 100, 150 mg/kg body weight in drug-treated animals. These results indicate that the protective effect of tea polyphenol was associated with inhibition of MDA induced by S180 cells and to maintain the antioxidant enzyme levels.

## 1. Introduction

Hepatocellular carcinoma (HCC), a leading cause of death in China and many Asian countries, is difficult to treat because of early progression and metastasis. It is well known that angiogenesis is essential for the survival, growth, and metastasis of tumor cells [1–5]. Hepatitis B (HBV) and Hepatitis C virus (HCV) are the risk factors attributed to 80% of HCC cases globally [6]. Cirrhosis, radiation, free radicals, genetic changes, metabolic disorder and exposure to certain chemical carcinogens such as aflatoxin, ethionine, diethylnitrosamine (DEN) and 2-acetylaminofluorene (AAF) have also been implicated in the pathogenesis of hepatocarcinoma [7–10].

Several human chronic disease states including cancer have been associated with oxidative stress produced through either an increased free radical generation and/or a decreased antioxidant level in the target cells and tissues [11–14]. A role for reactive oxygen radicals in the etiology of cancer is supported by epidemiologic studies. Specifically these epidemiologic studies illustrated the protective role for antioxidants against cancer development [15–17]. Continuous production of oxygen radicals leads to the formation of covalent bond adduct with DNA nucleic acid, which will subsequently result in mutagenicity. Endogenous antioxidant enzymes such as superoxide dismutase (SOD), glutathione peroxidase (GSH-Px) and catalase (CAT) have been reported to reduce the free radical formation and prevent oxidative damage [18,19].

Herbal medicines derived from plant extracts are being increasingly utilized to treat a wide variety of clinical disease [20]. More attention has been paid to the protective effects of natural antioxidants against drug-induced toxicities especially whenever free radical generation is involved [21,22]. Green tea and its constituent catechins are best known for their antioxidant properties, which has led to their evaluation in a number of diseases associated with reactive oxygen species (ROS), such as cancer, cardiovascular and neurodegenerative diseases. Several epidemiological studies as well as studies in animal models have shown that green tea can afford protection against various cancers such as those of the skin, breast, prostate and lung [23]. In addition to the cancer chemopreventive properties, green tea and EGCG have been shown to be anti-angiogenic (prevention of tumor blood vessel growth) [24,25] and anti-mutagenic [26,27]. Other health benefits attributed to green tea include antibacterial [28], anti-HIV [29], anti-aging [30] and anti-inflammatory activity [31].

In the present study, effect of tea polyphenol on oxidative injury in hepatocarcinoma mice was evaluated.

## 2. Results

### 2.1. Inhibitory Effects on the Tumor-Bearing Mice S180 Model by Tea Polyphenol

In an *in vitro* study, we found that tea polyphenol (20–200 μg/mL) could significantly inhibit S180 cell growth. Moreover, the inhibition rate was increased with increasing content of tea polyphenol (20–200 μg/mL).

There was no statistical significance in the mice’s body weights (beginning and end) between groups (*P* > 0.01, Table 1). Results showed that tea polyphenol had notable inhibitory effects on the sarcoma-loaded mice S180 model, which led to a depressed trend of tumor weights. The tumor inhibition rate of tea polyphenol at high dose, intermediate dose and low dose groups was 64, 48 and 28%, respectively (Table 2).

### 2.2. Effect of Tea Polyphenol on Serum AST, ALT and ALP Activities

Table 3 shows that serum AST, ALT and ALP activities in hepatocarcinoma control group (III) were significantly higher than those in normal control (group I). Compared with normal control (group I), administration of tea polyphenol (150 mg/kg body weight) decreased serum AST, ALT and ALP activities in group II, but there was no statistical difference in serum ALT and ALP. Administration of tea polyphenol (50, 100, 150 mg/kg body weight) dose-dependently significantly decreased serum AST, ALT and ALP activities in group IV, V and VI when compared to hepatocarcinoma control group (III).

### 2.3. Effect of Tea polyphenol on Serum WBC, TP, ALB and A/G

Table 4 shows that serum WBC, TP, ALB and A/G in hepatocarcinoma control group (III) were significantly lower than those in normal control (group I). Although administration of tea polyphenol (150 mg/kg body weight) significantly increased serum WBC, and TP in group II when compared to normal control group (I), the decrease in serum ALB and A/G was not significant. Administration of tea polyphenol (50, 100, 150 mg/kg body weight) dose-dependently significantly increased serum WBC, TP, ALB and A/G in group IV, V and VI when compared to hepatocarcinoma control group (III).

### 2.4. Effect of Tea Polyphenol on Serum TNF-α and IFN-γ

Table 5 shows the effect of tea polyphenol on serum TNF-α and IFN-γ in experimental mice. Results revealed a significant decrease in serum TNF-α and IFN-γ levels of hepatocarcinoma control group (III) compared with the normal control group (I). Administration of tea polyphenol (150 mg/kg body weight) significantly increased serum TNF-α and IFN-γ levels in group II when compared to normal control group (I). Administration of tea polyphenol (50, 100, 150 mg/kg body weight) dose-dependently significantly increased serum TNF-α and IFN-γ levels in group IV, V and VI when compared to hepatocarcinoma control group (III).

### 2.5. Effect of Tea Polyphenol on Liver MDA and GSH

Liver MDA concentration was significantly enhanced in hepatocarcinoma control group (III), whereas GSH level was markedly decreased compared to normal control group (I) (Table 6). Administration of tea polyphenol (150 mg/kg body weight) produced a significant decrease in liver MDA and increase in GSH level of group II mice in comparison with normal control group (I). The administration of tea polyphenol (50, 100, 150 mg/kg body weight) produced a significant decrease in liver MDA and increase in GSH level of group IV, V and VI mice. The decrease or increase was observed to be dose-dependent as a greater decrease or increase was observed in mice receiving 150 mg/kg tea polyphenol compared to those administered 100, 50 mg/kg tea polyphenol (Table 6).

### 2.6. Effect of Tea Polyphenol on Liver SOD, CAT, GSH-Px and GR Activities

Activities of liver SOD, CAT, GSH-Px and GR were significantly enhanced in group (II) compared to normal control group (I). Activities of liver SOD, CAT, GSH-Px and GR were significantly reduced in hepatocarcinoma control group (III) compared to normal control group (I). Administration of tea polyphenol at 50, 100, 150 mg/kg body weight dose-dependently significantly enhanced liver SOD, CAT, GSH-Px and GR activities in group IV, V and VI mice compared to hepatocarcinoma control group (III) (Table 7).

## 3. Discussion

In this study, we demonstrated that the implantation with S180 sarcoma to mice lead to a marked elevation in the levels of serum AST, ALT and ALP which is indicative of hepatocellular damage, as previously reported [32]. This elevation could potentially be attributed to the release of these enzymes from the cytoplasm into the blood circulation after rupture of the plasma membrane and cellular damage. Serum AST, ALT and ALP are biomarkers in the diagnosis of hepatic damage because they are released into the circulation after cellular damage [33]. Administration of tea polyphenol significantly reduced the activity of liver enzymes in blood of hepatocarcinoma mice (group III). Due to its ability to reduce free radical-induced oxidative damage in the liver, tea polyphenol has been shown to decrease liver enzymes in serum and prevent liver damage of rats with liver fibrosis [34]. Tea polyphenol prevents liver damage by maintaining the integrity of the plasma membrane, thereby suppressing the leakage of enzymes.

There are several different types of white cells in the blood in differing amounts. They all play a part in the immune response. A low white blood cell count (leukopenia) leaves your body more open to infection. Moreover, if an infection does develop, your body may be unable to fight it off. This is the response of the body to infection, or anything else the body recognizes as “foreign”. These blood cells can be made very quickly and generally have a short life. Some only live for a few hours, others for a few days [35]. Increased TP indicates dehydration or blood cancer, bone marrow cancer; decreases indicate malnutrition, poor digestion, liver or kidney disease, bleeding or burns [36,37]. ALB is produced by the liver, reduced levels of this protein can point to chronic liver or kidney disease, or parasitic infections such as hookworm. High levels indicate dehydration and loss of protein [38,39]. In the present study, serum WBC, TP, ALB and A/G in hepatocarcinoma control group (III) were significantly lower than those in normal control (group I). Increases in TP, A/G, albumin were observed in mice implanted with S180 sarcoma (group III), and the degrees of change were big, and related toxicological changes were evident. Administration of tea polyphenol (50, 100, 150 mg/kg body weight) dose-dependently significantly increased serum WBC, TP, ALB and A/G in group IV, V and VI when compared to hepatocarcinoma control group (III). This indicated that tea polyphenol can improve immunity function in hepatocarcinoma mice.

Tumor necrosis factor (TNF, cachexin or cachectin formerly known as tumor necrosis factor-alpha or TNF-α) is a cytokine involved in systemic inflammation and is a member of a group of cytokines that stimulate the acute phase reaction. It is produced chiefly by activated macrophages, although it can be produced by other cell types as well. The primary role of TNF is in the regulation of immune cells. TNF, being an endogenous pyrogen, is able to induce fever, to induce apoptotic cell death, to induce sepsis (through IL1 & IL6 production), to induce cachexia, induce inflammation, and to inhibit tumorigenesis and viral replication [40,41]. Most scholars now agree that TNF and IFN are related to liver damage. Furthermore, TNF-α increases the susceptibility of liver endothelial cells to Fas-mediated apoptosis [42]. Interferon-gamma (IFN-γ) is a dimerized soluble cytokine that is the only member of the type II class of interferons [43]. This interferon was originally called macrophage-activating factor, a term now used to describe a larger family of proteins to which IFN-γ belongs. A significant decrease in serum TNF-α and IFN-γ levels were observed in liver cancer control group (group III), suggesting that decreased serum TNF-α and IFN-γ levels was an important marker of liver cancer. Administration of tea polyphenol increased serum TNF-α and IFN-γ levels in mice implanted with S180 sarcoma (group IV–VI).

One strong mechanistic link between chronic inflammation and cancer is through the increased production of free radicals at the site of inflammation and the resulting molecular changes, which include lipid peroxidation and oxidative DNA damage [44]. Indeed, markers of DNA damage, such as 8-hydroxydeoxyguanosine (8-OHdG), and lipid peroxidation, such as 4-hydroxynonenal (HNE) and malondialdehyde (MDA), are commonly elevated in liver of patients with chronic hepatitis C virus infection and correlate well with the degree of viral infection and inflammation, known risk factors for Hepatocellular carcinoma [45]. Level of liver MDA was markedly increased, whereas activities of liver SOD, CAT, GSH-Px and GR were significantly reduced in mice implanted with S180 sarcoma (group III). This suggested that oxidative injury occurred in hepatocellular carcinoma animals.

It is well known that inflammation is one of the biological responses driven by oxidative stress. Modulation of oxidative damage as well as inflammation protect against hepatocarcinogenesis. It has been shown that resveratrol, a compound present in grapes and red wine, has potent antioxidant [46] and anti-inflammatory [47] properties, which might play an important role in protecting the liver against carcinogen-induced neoplasia. Recently, it was reported that resveratrol significantly prevents diethylnitrosamine (DENA)-induced liver tumorigenesis in rats [48]. Administration of tea polyphenol at 50, 100, 150 mg/kg body weight dose-dependently significantly enhanced liver SOD, CAT, GSH-Px and GR activities in experimental mice (group IV–VI). These results suggested that oxidative injury was related to hepatocellular carcinoma development, and tea polyphenol played its antitumor activity partly by decreasing oxidative injury and increasing antioxidant enzymes activity.

## 4. Experimental Section

### 4.1. Material

**Figure 1:**
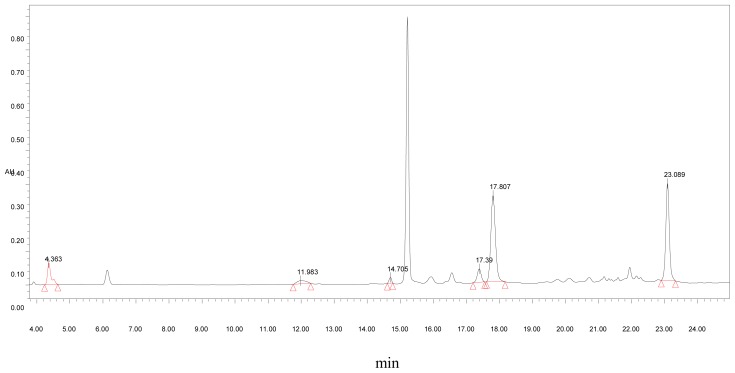
Chemical composition of tea polyphenol.

Tea polyphenol was purchased from Hangzhou HeTian TianBao Biotechnology Ltd. Its chemical composition was examined using HPLC (Table 1 and Figure 1).

### 4.2. Animals

C57BL/6J mice, aged 6–8 weeks and weighing 20–22 g, obtained from the microbiology Institute of Zhongshan University (Guangzhou, China), were used throughout the study. The animals were supplied with standard mouse pellet diet and water *ad libitum*.

### 4.3. Experimental Design

The effect of the tea polyphenol on tumor growth was estimated by evaluating body weight, tumor weight, and percentage of tumor inhibition. S180 tumor cell line was originally obtained from the microbiology Institute of Zhongshan University, and maintained as the ascites form by serial passages intraperitoneally in C57BL/6J mice. For solid tumor development, 0.2 mL of S180 cell suspension (2 × 10^7^ cells/mL) was inoculated subcutaneously into right armpits of mice under sterile condition. When tumor size was 1 cm, tumor tissues were removed and cut into 2 mm × 1 mm tumor mass. Then, mice were anesthetized and the abdomen was incised. The liver was exposed, and a hole was made in the left lobe of the liver by straight ophthalmic forceps (3 mm). Then, these tumor masses were placed into the holes in the mice livers. The mice were divided into five random groups (10 in each): S180-bearing control, normal control, and three tea polyphenol treatments (50, 100, 150 mg/kg body weight for group III, IV and V). Test doses were decided on the basis of findings from preliminary studies. Body weight of animals was recorded before the experiment. Tea polyphenol were orally administrated by gavage daily for 20 days. Normal control (group I) and S180-bearing control groups (II) received the same volume of normal saline. On the 20th day, all animals were executed. Their body and tumor weights were obtained and documented. Finally, the tumor inhibition rate was calculated.

### 4.4. Biochemical Analysis

AST, ALT, ALP, TNF-α, IFN-γ were measured with commercially available Kits.

White blood cell (WBC) count was measured using a hematological autoanalyzer (Advia 120E, Bayer, Newbury, Berkshire, UK). Blood collected for serum biochemistry was untreated and centrifuged at 3000 rpm for 10 min. The serum obtained was examined for the following parameters using a biochemistry autoanalyzer (Hitachi 7060, Tokyo, Japan): total protein (TP), albumin (ALB), A/G ratio.

Liver MDA concentration was determined by using the method described by Draper and Hadley [49,50] based on TBA reactivity. Briefly, 2.5 mL of 10% trichloracetic acid and 0.5 mL of homogenate were added into tubes and mixed. After incubating for 15 min at 90 °C and cooling with cold water the mixture was centrifuged at 3000 rpm for 10 min. Two milliliters of supernatant were taken and 1ml of 0.675% TBA was added. The tubes were sealed and incubated at 90 °C for 15 min and then cooled to room temperature. The optical density was measured at 532 nm by a spectrophotometer.

Determination of reduced glutathione level (GSH) in liver was carried out according to the previously described colorimetric method [51]. This method depends on the reaction of reduced glutathione with 5,5-dithiobis, 2-(nitrobenzoic acid) that can be measured colorimetrically. The yellow color developed was measured at 412 nm against a blank reagent.

Superoxide dismutase (SOD) activity in liver homogenate was determined according to the method of Minami and Yoshikawa [52]. This method is based on the generation of superoxide anions by pyrogallol autoxidation, detection of generated superoxide anions by nitro blue tetrazolium (NBT) formazan color development and measurement of the amount of generated superoxide anions scavenged by SOD (the inhibitory level of formazan color development). The homogenate was centrifuged at 105,000 g for 15 min at 4 °C. To 0.25 mL of supernatant, 0.5 mL of tris cacodylic buffer, 0.1 mL of 16% triton x-100 and 0.25 mL NBT were added. The reaction was started by the addition of 0.01 mL diluted pyrogallol. Incubation was maintained for 5 min at 37 °C. The reaction was stopped by the addition of 0.3 mL of 2 M formic acid. The formazan color developed was determined spectrophotometrically (Spectronic 501, Shimadzu).

Catalase activity of tissues was measured by the method of Aebi [53]. To a cuvette containing 1.9 mL of 50 mmol phosphate buffer (pH 7.0), the appropriate dilution of tissue supernatant was added. The final volume of the mixture was made up to 2 mL by adding additional buffer solution. The reaction was started by the addition of 1.0 mL of freshly prepared 30 mmol H_2_O_2_. The rate of decomposition of H_2_O_2_ was measured spectrophotometrically at 240 nm.

The method of Lawrence and Burk [54] as described by Mantha *et al*. [55] was used to measure GSH-Px activity. The assay mixture consisted of 2.0 mL of 75 mmol phosphate buffer (pH 7.0), 50 μL of 60 mmol glutathione, 0.1 mL of 30 units/mL glutathione reductase, 0.1 mL of 15 mmol disodium salt of EDTA, 0.1 mL of 3 mmol reduced NADPH and an appropriate amount of tissue supernatant to a final volume of 3.0 mL. The reaction was started by addition of 0.1 mL of 7.5 mmol H_2_O_2_. The rate of change of absorbance during the conversion of NADPH to NADP^+^ was recorded spectrophotometerically at 340 nm for 3 min. GSH-Px activity was expressed as μmol of NADPH oxidized to NADP^+^ min^−1^ (mg^−1^ protein) using an extinction coefficient (6.22 mM^−1^·cm^−1^) for NADPH. Protein content of the samples was determined by the Biuret method of Gornall *et al*. [56].

Glutathione reductase activity was assayed as described by Calberg and Mannervik [57], with some modifications, by measuring the oxidation of NADPH at 340 nm. The reaction mixture consisted of 0.1 M sodium phosphate buffer (pH 7.5), 1 mM EDTA, 0.63 mM NADPH and 0.15 mM GSSG.

### 4.5. Statistical Analysis

Data was statistically evaluated using one-way ANOVA in SPSS for Windows (version 11.5; SPSS Inc.: Chicago, IL, USA, 2002) installed computer. The values were considered significant when (*P* < 0.05).

## 5. Conclusions

Mice implanted with S180 sarcoma showed a significant increase in serum AST, ALT and ALP activities, liver MDA level, decrease in serum WBC, TP, ALB, A/G, TNF-α and IFN-γ, and liver GSH, SOD, CAT, GSH-Px and GR activities. This indicated that in hepatocarcinoma mice, liver function, and immunity activity have been greatly weakened and decreased. In addition, oxidative injury has been increased with decreased antioxidant enzymes activity. Twenty days of tea polyphenol treatment significantly decreased serum AST, ALT and ALP activities, liver MDA level, increase serum WBC, TP, ALB, A/G, TNF-α and IFN-γ, and liver GSH, SOD, CAT, GSH-Px and GR activities. These results showed that tea polyphenol can improve liver function, and immunity activity, decrease oxidative injury in hepatocarcinoma mice implanted with S180 sarcoma. Our present work indicated that tea polyphenol has a potential liver protection effect. It will be our necessary work in the future to test pharmacological function of individual components in the tea polyphenol in hepatocellular carcinoma animals.

## Figures and Tables

**Table 1:** High Performance Liquid Chromatography analysis of tea polyphenol.

Sample	Chemical Components	Retention Time (min)	Peak Area	Content (mg/g)
Tea polyphenol	GA	4.363	441,213.5	8.05
	EGC	11.984	216,448.9	52.85
	C	14.705	83,710.41	6.51
	EC	17.390	283,894.6	22.63
	EGCG	17.807	2,378,683	88.87
	ECG	23.089	2,110,191	65.09
	catechin	-	-	235.94

**Table 2:** Inhibitory effects on the tumor-bearing mice S180 model by tea polyphenol.

Group	Body Weight (g)		Tumour Weight (g)	Inhibition Rate (%)
	
	Begin	End		
I	21.42 ± 1.76	27.46 ± 1.63	-	-
II	21.53 ± 1.59	27.22 ± 1.72	-	-
III	21.85 ± 1.88	26.98 ± 2.07	0.25 ± 0.01	-
IV	20.97 ± 1.37	26.83 ± 2.14	0.18 ± 0.01	28
V	22.07 ± 1.59	26.79 ± 1.99	0.13 ± 0.02	48
VI	21.68 ± 1.60	27.15 ± 1.94	0.09 ± 0.01	64

**Table 3:** Effect of tea polyphenol on serum serum aspartate transaminase (AST), alanine aminotransfere (ALT) and alkaline phosphatase (ALP) activities.

Group	AST (U/L)	ALP (U/L)	ALT (U/L)
I	248.47 ± 13.86	114.18 ± 8.65	77.38 ± 3.76
II	225.91 ± 17.11 a	108.95 ± 9.37	70.28 ± 4.71
III	307.21 ± 21.65 b	134.26 ± 9.04 b	94.18 ± 4.88 b
IV	278.41 ± 16.37 d	125.03 ± 6.83 c	88.02 ± 3.97 c
V	266.58 ± 18.38 d	121.55 ± 7.93 c	82.15 ± 4.17 d
VI	259.03 ± 19.47 d	117.03 ± 9.03 d	79.41 ± 2.86 d

a *P* < 0.05;

b *P* < 0.01, compared with group I;

c *P* < 0.05;

d *P* < 0.01, compared with group III.

**Table 4:** Effect of tea polyphenol on serum white blood cells (WBC), total protein (TP), albumin (ALB) and A/G.

Group	WBC (×10^9^/L)	TP (g/L)	ALB (g/L)	A/G
I	23.05 ± 1.32	59.52 ± 1.86	33.41 ± 1.42	1.59 ± 0.09
	24.91 ± 1.95 a	61.02 ± 2.93 a	34.24 ± 1.66	1.63 ± 0.11
II	17.31 ± 1.11 b	55.27 ± 1.95 b	31.54 ± 1.76 b	1.09 ± 0.1 b
III	19.33 ± 1.43 c	57.21 ± 1.78 c	31.95 ± 1.48	1.15 ± 0.08
IV	21.49 ± 1.76 c	58.72 ± 2.31 c	32.41 ± 1.33	1.24 ± 0.09 c
V	22.42 ± 1.28 d	58.83 ± 2.41 c	32.77 ± 1.28 c	1.44 ± 1.05 d

a *P* < 0.01;

b *P* < 0.01, compared with group I;

c *P* < 0.05;

d *P* < 0.01, compared with group III.

**Table 5:** Effect of tea polyphenol on serum tumor necrosis factor-alpha (TNF-α) and Interferon-gamma (IFN-γ).

Group	TNF-α (pg/mL)	IFN-γ (pg/mL)
I	839.42 ± 32.18	505.37 ± 21.53
II	873.67 ± 38.63 b	518.09 ± 32.85
III	401.65 ± 18.39 b	251.73 ± 16.31 b
IV	548.29 ± 20.61 d	332.84 ± 11.66 d
V	648.11 ± 22.17 d	428.91 ± 19.06 d
VI	782.61 ± 19.62 d	489.74 ± 21.64 d

b *P* < 0.01, compared with group I;

d *P* < 0.01, compared with group III.

**Table 6:** Effect of tea polyphenol on liver malondialdehyde (MDA) and glutathione (GSH).

Group	MDA (nmol/mg prot)	GSH (nmol/mg)
I	2.76 ± 0.13	63.17 ± 3.11
II	1.59 ± 0.18 b	86.64 ± 6.53 b
III	8.94 ± 0.42 b	34.28 ± 1.53 b
IV	5.37 ± 0.17 d	48.29 ± 1.85 d
V	4.48 ± 0.19 d	56.03 ± 2.31 d
VI	3.51 ± 0.18 d	60.41 ± 2.65 d

b *P* < 0.01, compared with group I;

d *P* < 0.01, compared with group III.

**Table 7:** Effect of tea polyphenol on liver superoxide dismutase (SOD), catalase (CAT), glutathione peroxidase (GSH-Px) and GR activities.

Group	SOD (U/mg prot)	CAT (U/mg prot)	GSH-Px (U/mg prot)	GR (U/mg prot)
I	362.81 ± 11.43	54.19 ± 2.06	48.22 ± 1.63	33.15 ± 1.09
II	414.59 ± 15.22 b	71.43 ± 3.08 b	69.27 ± 2.25 b	49.54 ± 1.65 b
III	148.25 ± 10.63 b	23.18 ± 1.11 b	20.75 ± 1.33 b	14.28 ± 1.21 b
IV	195.39 ± 12.64 d	37.08 ± 1.74 d	33.15 ± 1.58 d	21.14 ± 1.31 d
V	257.31 ± 15.03 d	42.61 ± 2.31 d	39.92 ± 1.76 d	27.05 ± 1.48 d
VI	342.08 ± 17.22 d	51.29 ± 2.65 d	48.13 ± 1.92 d	31.96 ± 1.39 d

b *P* < 0.01, compared with group I;

d *P* < 0.01, compared with group III.
